# Implementation of a Multi-Disciplinary Geriatric Oncology Clinic in Toronto, Canada

**DOI:** 10.3390/curroncol32020089

**Published:** 2025-02-06

**Authors:** Ines B. Menjak, Khloe Campos, Mark Pasetka, Arlene Budden, Elaine Curle, Leslie Gibson, Ewa Szumacher, Rajin Mehta

**Affiliations:** 1 Department of Medicine, University of Toronto, Toronto, ON M5S 1A1, Canada; 2 Department of Medical Oncology and Malignant Hematology, Sunnybrook Health Sciences Centre, Toronto, ON M4N 3M5, Canada; 3 Department of Evaluative Clinical Sciences, Sunnybrook Research Institute, Toronto, ON M4N 3M5, Canada; 4 Department of Psychology, University of Toronto, Toronto, ON M5S 1A1, Canada; 5 Department of Pharmacy, Sunnybrook Health Sciences Centre, Toronto, ON M4N 3M5, Canada; 6 Department of Nursing, Sunnybrook Health Sciences Centre, Toronto, ON M4N 3M5, Canada; 7 Department of Occupational Therapy, Sunnybrook Health Sciences Centre, Toronto, ON M4N 3M5, Canada; 8 Department of Radiation Oncology, Sunnybrook Health Sciences Centre, Toronto, ON M4N 3M5, Canada; 9 Department of Radiation Oncology, University of Toronto, Toronto, ON M5S 1A1, Canada; 10 Department of Geriatric Medicine, Sunnybrook Health Sciences Centre, Toronto, ON M4N 3M5, Canada

**Keywords:** geriatric, oncology, geriatric assessment, decision-making, geriatric oncology clinic, allied health

## Abstract

Older adults with cancer tend to face more complex health needs than their younger counterparts. Patients > 65 years of age are recommended for comprehensive geriatric assessment (CGA) to capture and address age-related vulnerabilities. Access to geriatrics services is limited, and our baseline audit of geriatric referrals in 2019 from the cancer program revealed that only 30% of patients referred received a CGA. The aim of this study was to assess the implementation of a geriatric oncology (GO) clinic that employs CGA and determine patient outcomes. We conducted a retrospective cohort study at a single institution. Data collection included baseline characteristics, GO clinic findings and characteristics, recommendations/referrals, and emergency room (ER) visits/hospitalizations within 6 months of CGA. Descriptive statistics were used for analysis. A total of 100 patients were included, with a median (range) age of 80 (63–97) years; 70% were female, and the most common cancer type was breast (31%). Through the GO clinic, patients were seen in a timely manner, with a median of 3 weeks, compared to our historical baseline of 11 weeks. Cognitive decline (32%) and pre-treatment CGA (22%) were the most common reasons for referral, and the most common new diagnosis was cognitive impairment (65%). For pre-treatment CGA, 16 (48%) patients were deemed suitable for treatment and 10 (30%) were recommended for modified treatment; 34 (94%) referring physicians followed the recommendation. In addition, most (68%) patients received an allied health referral. One third of patients visited the ER and 30 (30%) patients were hospitalized. Overall, the GO clinic resulted in greater access to CGA in a timely manner, enhanced access to allied health, and assisted in treatment decision-making.

## 1. Introduction

A great majority of patients with cancer are older adults. By 2030, it is estimated that about 70% of cancer diagnoses will be represented by patients 65 years and older [1]. With aging, major changes can occur regarding one’s health, functioning, mental status and social support [2]. These changes can influence how patients respond to cancer treatment [3]. Due to age-related vulnerabilities and conditions, older adults tend to face more challenges and toxicities with cancer treatment [4].

Although older adults constitute more than half of the population of patients diagnosed with cancer, they are underrepresented in clinical trials that study the effects of various cancer treatments [5]. In a study performed by Talarico et al., researchers found that only 36% of patients registered for new cancer therapy trials in the United States were over the age of 65 [6]. Due to limited evidence regarding suitable treatment, it is not always appropriate to apply the results of clinical trials to older adults seeking treatment. With advancing age, the needs of patients with cancer become much more complex, and many trials to date do not consider age-related vulnerabilities [7,8,9]. According to the Centers for Disease Control and Prevention, about 85.6% of older adults have one or more chronic conditions, and approximately 56.0% have two or more [10]. Older patients with cancer are specifically at a higher risk of toxicity and post-operative complications due to co-morbidities, frailty, polypharmacy, geriatric issues, and physiological changes [11,12].

The lack of research on older patients with cancer makes it more difficult create treatment plans and provide management tools that are appropriate to capture patient needs. According to ASCO guidelines, a geriatric assessment should be performed to capture potential vulnerabilities and impairments that would not be typically identified in standard oncology care assessments [13]. The comprehensive geriatric assessment (CGA) provides a diverse approach and solution to this knowledge gap. A CGA can provide essential information that is useful in creating a personalized, coordinated and integrated treatment plan to potentially improve the care of older patients with cancer. It can identify unrecognized health problems that have the potential to impact patients’ cancer treatment; according to a review conducted by Caillet et al., CGA can influence approximately 21–49% of treatment decisions [14]. It is a multidisciplinary process that determines an older individual’s medical, psychosocial and functional history and current issues, creating a linkage between medical and social care [15]. It seeks to provide a structured comprehensive assessment that works towards patient-centered goals [16].

Various studies have provided insight into the significance and benefits of implementing a CGA when considering cancer treatment plans for older adults. According to Extermann et al., there is strong evidence to suggest that a CGA can detect many unknown problems commonly missed in standard oncology appointments [17]. Older adults are at a higher risk of experiencing grade 3 or higher chemotherapy-related toxic effects [18]. Two recent randomized clinical trials examined the impact of geriatric assessment on cancer treatment-related toxic effects [18,19]. In both studies, researchers found that the integration of geriatric assessment significantly reduced serious chemotherapy-related toxic effects when compared to patients who received standard oncology care. Its implementation has also been shown to improve overall function, survival rates, and treatment completion, with fewer required modifications, and reduce hospitalizations, emergency room (ER) visits and costs [17,20]. Overall, CGA provides value in identifying issues otherwise not detected, augments non-oncologic domains, influences chemotherapy intensity and may improve treatment completion and chemotherapy tolerance.

The Sunnybrook Health Sciences Centre (SHSC)’s Odette Cancer Centre is one of five health centers in Canada that have a geriatric oncology (GO) clinic implementing CGA. The GO clinic was developed to provide better access to geriatric assessment for patients with cancer. In 2019, a baseline audit revealed that only 30% of oncology patients referred to the general geriatrics clinic received a consultation, with an average time to consultation of 11 weeks.

The aims of the GO clinic are to increase access to CGA, reduce the referral time for geriatrics assessment, assist in decision-making for cancer treatment, address complex health needs of older patients and enhance access to allied health supports. The primary objective of this study was to review the implementation of the GO clinic and to assess whether it enabled a greater proportion of patients to access geriatric care and reduced the time to consultation compared to our baseline audit.

## 2. Materials and Methods

This was a retrospective cohort study at a single institution conducted between 12 March 2020 and 29 February 2024. There was a period of reduced clinical activity due to COVID-19, as mandated by our institution, from approximately March to July 2020. There was an additional period of clinic closure due to staffing issues in October–December 2021. Patients are eligible to be seen in the GO clinic if they are 65 years or older and known to an oncologist at our institution.

Data were extracted from patient electronic medical records and the electronic referral system. The data collected included patients’ baseline characteristics, cancer treatment status, findings and recommendations from the initial and follow-up consultations for the CGA, as well as ER visits and hospitalizations at SHSC for six months after the CGA.

This was a quality improvement study to review the implementation of the multidisciplinary GO clinic. The primary outcome measures were the proportion of patients referred to geriatrics that received a consultation, as well as the time to consultation. These were compared to the historical control benchmark established from a baseline audit of data from the year prior to the clinic’s inception.

Descriptive statistics were used to analyze the data with Microsoft Excel 2016. The descriptive statistics used included measures of frequency and central tendency, such as the mean, median, standard deviation, range or interquartile range, when appropriate. This project received research ethics board approval and Quality Improvement Project approval through the Department of Quality and Patient Safety at our institution.

### Clinic Description

The GO clinic is a multidisciplinary clinic that follows a multiple-step process involving different providers to conduct a comprehensive assessment. The clinic providers include a pharmacist, nurse, or occupational therapist (OT), a trainee physician, medical oncologist and geriatrician. Eligible patients are triaged based on the referring physician’s reason for referral and the urgency of the request. Priority is given to patients who require input on treatment, followed by patients with geriatric issues during cancer treatment, followed by patients who are on surveillance. Due to limited resources, the GO clinic is available once a week for one new patient consultation and two follow-ups.

The GO clinic includes phone pre-assessments that are performed within a week before CGA. Pre-assessments include pharmacy and OT or nursing. The pharmacist reviews the patient’s medications, including their packaging, administration and understanding of the medications, and makes relevant recommendations for the physicians. OT and nursing conduct a functional assessment, a geriatric review of systems, and use the Geriatric Depression Scale (GDS) [21]. Nursing pre-assessments were implemented in September of 2022 to replace OT pre-assessments due to a lack of OT resources. The in-person CGA is typically conducted by a trainee physician, and the trainee physician, geriatrician and medical oncologist review the consultation and develop the plan together.

The CGA consists of standard components including medical history, active issues, treatment status, social history, a geriatric review of systems, cognitive, mental and functional assessments, and a physical exam. The geriatric review of systems includes an assessment of mental status history, fall risk, incontinence, pain, skin issues, sensory impairments, communication difficulties, nutrition status, sleep disturbances, alcohol use, smoking, safety issues and caregiver issues. The assessment also includes a set of validated tests and classifications: GDS [21], the modified Katz Index of Independence in Activities of Daily Living (ADL) [22], the modified Lawton Instrumental Activities of Daily Living (IADL) Scale [23], the Rockwood Clinical Frailty Scale [24] and the Cancer and Aging Research Group (CARG) Chemotherapy Toxicity Tool when applicable [25]. Cognitive test selection is at the physician’s discretion and includes one of the following: the Montreal Cognitive Assessment (MOCA) [26], the Mini-Mental State Examination (MMSE) [27], the Rowland Universal Dementia Assessment Scale (RUDAS) [28], or the Mini-Cog [29]. In general, the Mini-Cog was only performed on a pilot basis for a small number of patients and is not part of routine care. In the clinic’s modified version of the Katz Index of Independence in ADL, the categories include feeding, dressing, bathing, toileting, ambulation and transfers. The modified version of the Lawton IADL Scale considers the following categories: driving/near accidents, shopping, meal preparation, housework, laundry and banking. In the clinic’s modified version of the Lawton IADL Scale, potential gender bias is not considered. In addition, in the analysis, if the patient has never performed one of the IADL categories, it is not considered a deficit. For example, if the patient has never driven, they would obtain a point for that category.

The clinic was not intended for the long-term follow-up of geriatric issues. If the patient required long-term follow-up, the geriatrician affiliated with the GO clinic continued follow-up in the general geriatrics clinic affiliated with SHSC.

## 3. Results

### 3.1. Baseline Characteristics

A total of 100 patients were eligible for inclusion in this study. The patients’ baseline characteristics are outlined in Table 1. The patient population had a median (range) age of 80 (63–97), with the majority being female (70%). The most common cancer types were breast (33 [31%]), gastrointestinal (27 [25%]), and skin malignancies (14 [13%]), and 49% of patients had early-stage cancer, followed by 46% with advanced-stage cancer.

### 3.2. Geriatric Oncology Clinic Characteristics, Findings and Recommendations

All geriatric oncology clinic findings and recommendations are outlined in Table 2. Most CGAs were conducted in person (89 [89%]), with only a few virtual visits (phone [8%] or video call [3%]), mainly due to the COVID-19 pandemic. Referring physicians most commonly requested that patients were seen within two weeks of the referral (35 [35%]) or at the next available appointment (27 [27%]). Other urgency requests included less than one week (18 [18%]), within 3–4 weeks (4 [4%]) and within 5–6 weeks (16 [16%]). A sensitivity analysis was conducted to determine the number of patients seen within the target requested to exclude patients who were not seen within the target due to the patient/family rebooking the appointment or clinic closure. In the sensitivity analysis, more than half of the patients were seen within the target requested (56%). The median (range) number of visits to the GO clinic was 2 (1–5) and the median (range) number of visits to the general geriatrics clinic for long-term follow-up was 0 (0–5).

**Figure 1:**
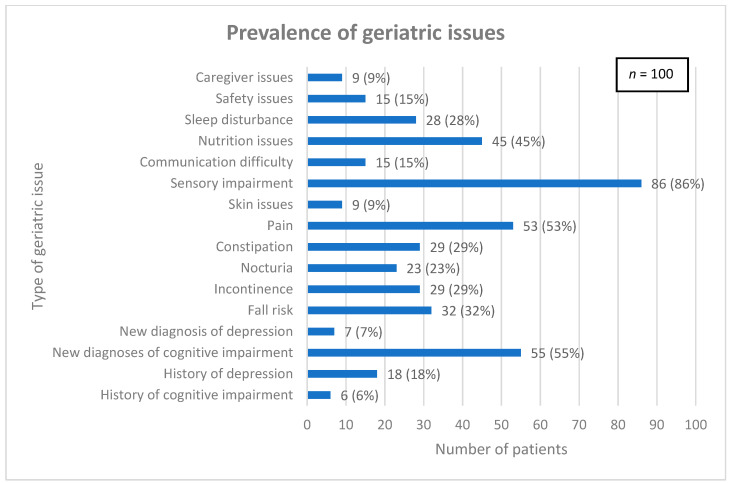
Prevalence of geriatric issues among patient population.

Common geriatric issues were assessed and are detailed in Figure 1. The median (interquartile range) total number of geriatric issues per patient was 4 (3, 6).

Regarding cognitive outcomes, most patients (83%) completed a cognitive test: MOCA (60 [72%]), RUDAS (16 [19%]), MMSE (5 [6%]) and Mini-Cog (2 [2%]). Patients did not complete a cognitive test due to reasons including the patient/family’s refusal (6 [35%]), the patient discontinuing a partially completed test (2 [12%]), and unknown (9 [53%]). Most patients (53%) had a positive (abnormal) cognitive screen.

**Figure 2:**
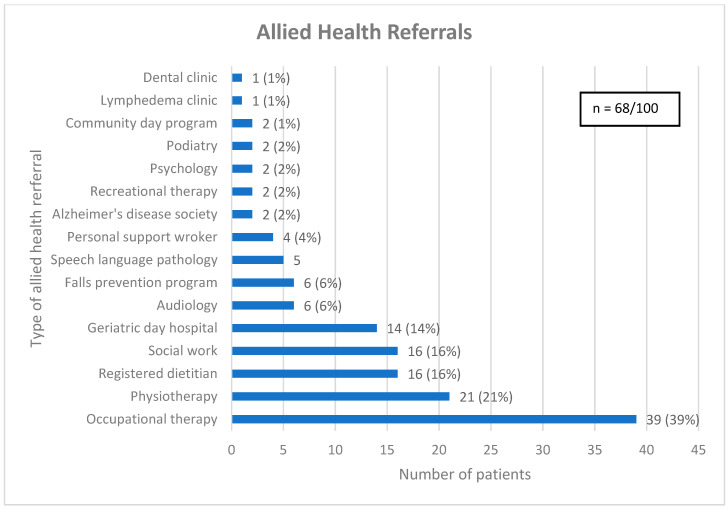
Referrals for allied health. Legend: Community day program = A program aimed at older adults with cognitive impairment, designed to optimize cognitive health through lifestyle modifications, memory training and psychosocial support. Geriatric day hospital = Consists of a geriatrician, nurse clinician, occupational therapist, physiotherapist, recreation therapist, social worker, and speech language pathologist.

As a result of CGA, 65 (65%) patients received one or more new diagnoses and the median (interquartile range) number of diagnoses given per patient was 1 (0, 1). Most patients (68%) received one or more allied health referrals, with the median (interquartile range) number of allied health referrals given per patient being 1 (0, 2) (see Figure 2). Patients who were at risk of falls were often referred for physiotherapy (31%), while others were referred to the falls prevention program (16%) or geriatric day hospital (28%), both of which have an embedded physiotherapist. Some patients did not receive an allied health referral due to seeing a related allied health professional prior to CGA (13%). Similarly, nutrition issues were commonly addressed with a referral to a registered dietitian (RD) (29%), a speech language pathologist (SLP) (11%) or the geriatric day hospital (18%), which has an SLP, with several patients seeing a related allied health professional before CGA (27%).

### 3.3. Cancer Treatment Status and Characteristics Post-CGA

The details of the patients’ oncology treatment status are presented in Table 3. Of the 33 patients that were referred for treatment post-CGA, 16 (48%) were deemed suitable for treatment, 10 (30%) were recommended for a modified or dose-reduced treatment and 7 (21%) were not recommended for treatment. Thirty one (94%) of the referring physicians followed through with the recommendation.

### 3.4. ER Visits and Hospitalizations

Details of the patients’ ER visits and hospitalizations are listed in Table 4. Approximately one-third of the patients had an ER visit (33 [33%]) or hospitalization (30 [30%]) within 6 months of CGA. There were seven patients with multiple ER visits and eight patients with multiple hospitalizations.

## 4. Discussion

In this retrospective analysis of the first 100 patients seen in the multidisciplinary GO clinic, it is clear that the GO clinic has achieved its goals of improving access to geriatric assessment, reducing the referral time, assisting in decision-making for cancer treatment, addressing the complex health needs of older patients, and increasing access to allied health. Access to CGA for older patients at our center increased in comparison to the historical approach of the general geriatric consultation service, with the proportion of referred patients receiving CGA increasing from 30% (17/55) in 2019 to 97% (100/103) with the GO clinic. Similarly, the average time from referral to consultation decreased from 11 weeks to approximately 3 weeks. In terms of cancer treatment decision-making, a third of patients were referred for pre-treatment CGA, where major concerns that potentially affected the course of care were addressed. The complex needs of older oncology patients were addressed, as 65 new comorbid diagnoses were made, one fifth of patients received one to two specialist referrals and almost half had medication changes. Access to allied health was enhanced, with over two-thirds of patients receiving one or more allied health referrals.

The results demonstrated in this study are congruent with the current literature on CGA in oncology. CGA can provide oncologists with recommendations and knowledge to assist in decision-making for optimal treatment plans for older patients. Two randomized controlled trials highlighted the positive effects of an assessment that tailors treatment to older patients and found that the implementation of a geriatric assessment significantly reduced chemotherapy-related toxicity [18,19]. In addition, in two similar retrospective reviews of geriatric oncology clinics in Mexico and France, researchers examined the impact of a geriatric oncology clinic on treatment plans [30,31]. In both studies, there was high adherence among oncologists to the changes to the treatment plan suggested by the geriatric oncology assessment. Similarly, our results found that among the 33 patients who received a recommendation for treatment, 94% of referring physicians followed through with the recommendation. These findings support the value of CGA in treatment decision-making.

Although the GO clinic was open to all types of cancer, it is evident that some types were more represented than others, and despite being common cancer types, there were fewer patients with lung and prostate cancer represented. Possible explanations for this could include variations in the comfort of the provider in managing older patients, the relative complexity of patients, patients being agreeable to the referral, a different perceived risk–benefit ratio for treatment, or a poorer prognosis in patients who may be more symptomatic from their cancer and thus require a different focus, i.e. palliative care.

Maintaining independence is important among older patients [32]; therefore, to maintain independence and allow better tolerance to cancer treatment, patients may benefit from allied health support and recommendations. The implementation of CGA is necessary to identify the allied health needs and concerns of older adults as it captures health complexities and vulnerabilities [33]. In a study conducted by Garric et al., researchers examined the impact of CGA on older adults with hematological malignancies who were considering treatment [34]. They found that the factors captured by CGA, such as functional and mobility impairment, are associated with a change in treatment plan. Therefore, optimizing functional limitations and mobility via allied health can have an impact on patient’s treatment plans and tolerability. Although our patient population mostly consisted of patients who could independently perform ADLs, and the median Rockwood CFS was 5 (mildly frail), we identified many vulnerable patients and provided several allied health referrals and recommendations to the majority of patients.

Cognitive impairment was the number one reason for referral to the GO clinic (32%), as well as the most common diagnosis made after CGA (65%). Our clinic used multiple cognitive screening tools, and more than half of patients had abnormal cognitive screening. Issues related to cognition are of particular concern among older patients with cancer when considering cancer treatments and toxicity. Cognitive function may be negatively affected by cancer and cancer treatment, and older adults with pre-existing impairments in cognition may be more susceptible to treatment-related adverse events [35]. This highlights the importance of implementing cognitive screening tests in routine oncology appointments, particularly among vulnerable populations.

Our clinic is one of very few GO clinics in Canada, and to our knowledge, the only clinic in Canada that utilizes a shared consultation with a medical oncologist and geriatrician. Pre-assessments were implemented to ensure that any concerns regarding medications or functional status were addressed before CGA, and to improve the efficiency of the in-person component given our limited geriatric resources. Collaboration between the medical oncologist and geriatrician allowed for clear communication in real time to create tailored treatment plans and appropriate recommendations. The embedded nurse and other allied health professionals assisted in operationalizing the recommendations. As it is uncommon for a GO clinic to have a medical oncologist other than the patient’s primary oncologist in addition to a geriatrician, further research into resource utilization will be valuable. Furthermore, methods that utilize nursing or other staff to follow-up with patients to ensure recommendations are carried out is another solution being considered during the ongoing evolution of this clinic.

There are limitations to this study, one of which is the relatively small sample size. However, as this is a single-center pilot clinic and CGA is a resource-intensive assessment, it was not possible to add additional patients in a feasible timeframe. Another limitation of this study is that it was conducted at a single tertiary academic cancer center. The population of patients in this catchment tend to have a higher education and socioeconomic status than the overall population. Lastly, studies with a retrospective design have inherent limitations; however, there were very few patients with missing data.

## 5. Conclusions

This retrospective review demonstrated that a geriatric oncology clinic improved access to geriatric assessment, and reduced the referral time to geriatric services compared to our baseline audit. In addition, the GO clinic assisted physicians in cancer treatment decision-making, addressed complex health needs and enhanced access to allied health. The unique features of this clinic included multidisciplinary pre-assessments, and a medical oncologist and geriatrician reviewing each consultation together to create a comprehensive plan for each patient. This study adds to the growing literature that supports the use of specialized geriatrics services for older patients with cancer, who are indeed a vulnerable population with complex needs. Future directions include prospective data collection in a larger population, as well as a study of resource utilization.

## Figures and Tables

**Table 1:** Baseline characteristics.

	Total
	(*n* = 100)
Age	
Median (Range)	80 (63–97)
Sex	
Female	70 (70%)
Male	30 (30%)
Cancer type (*n* = 106) ^a^	
Breast	33 (31%)
Gastrointestinal	27 (25%)
Skin	14 (13%)
Lung	10 (9%)
Malignant hematology	8 (8%)
Genitourinary	8 (8%)
Gynecologic	3 (3%)
Head and neck	3 (3%)
Cancer stage (*n* = 106) ^a^	
Early stage (I-III)	52 (49%)
Advanced stage (IV)	49 (46%)
No proven cancer—IPMN	3 (3%)
N/A—Suspected MDS or MDS/MPN overlap	2 (2%)
Number of active and past medical conditions	
Median (Interquartile range)	7 (5, 10)
Pre-existing conditions of interest	
Depression	18 (18%)
Delirium	9 (9%)
Dementia/cognitive impairment	6 (6%)
Number of current medications	
Median (Interquartile range)	7 (4, 10)
Cancer treatment status at the time of CGA	
On treatment ^b^	41 (41%)
Considering treatment	29 (29%)
On surveillance	25 (25%)
Current treatment on hold	5 (5%)
Type of cancer treatment at time of CGA (*n* = 52) ^c^	
Endocrine therapy	21 (40%)
Chemotherapy	16 (31%)
Immunotherapy	10 (19%)
Targeted therapy	4 (8%)
Radiation	1 (2%)
Dosing of current treatment (*n* = 46)	
Ideal dose	27 (59%)
Reduced	16 (35%)
Increased	2 (4%)
N/A	1 (2%)

Abbreviations: IPMN, intraductal papillary mucinous neoplasm; MDS, myelodysplastic syndrome; MPN, myeloproliferative neoplasms; CGA, comprehensive geriatric assessment; ^a^ 6/100 patients had two cancer types; ^b^ 4 patients that were on treatment were also considering another type of treatment, however, this is not evident in the “Considering treatment” numerical summary; ^c^ 6/46 patients on/on hold for treatment received two types of treatment.

**Table 2:** Geriatric oncology clinic characteristics, findings and recommendations.

	Total
	(*n* = 100)
Proportion of patients seen	100/103 (97%)
Time frame seen, days	
Median (Interquartile range)	21 (14, 33)
Sensitivity Analysis—Time frame seen, days (*n* = 94) ^d^	
Median (Interquartile range)	18 (13, 31)
Treatment candidate patients—Time frame seen, days (*n* = 33)	
Median (Interquartile range)	15 (10, 18)
Patients seen within target requested	54 (54%)
Reasons not seen within target (*n* = 46)	
Earliest slot available	35 (76%)
Clinic understaffed	4 (9%)
Patient/patient’s family requested to rebook	2 (4%)
Clinic closure	2 (4%)
Delayed due to awaiting investigations	1 (2%)
Administrative delay	1 (2%)
Unknown	1 (2%)
Specialty of referring physician	
Medical oncology	77 (77%)
Surgery	13 (13%)
Radiation oncology	6 (6%)
Malignant hematology	3 (3%)
Palliative medicine	1 (1%)
Reasons for referral (*n* = 150) ^e^	
Cognitive decline	48 (32%)
Treatment candidate	33 (22%)
Functional decline/Frailty	22 (15%)
Mobility/Falls	19 (13%)
Multi-morbidity	8 (5%)
Polypharmacy	5 (3%)
Mood/Behaviour	3 (2%)
Medical optimization prior to surgery	2 (1%)
General assessment	2 (1%)
Other ^f^	8 (5%)
Pre-assessments	
Pharmacy (*n* = 100)	86 (86%)
Occupational therapy (*n* = 54) ^g^	34 (63%)
Nursing (*n* = 46) ^g^	36 (78%)
Abnormal cognitive screen result	
MOCA (*n* = 60)	46 (77%)
RUDAS (*n* = 16)	5 (31%)
MMSE (*n* = 5)	2 (40%)
Mini-Cog (*n* = 2)	0 (00%)
Abnormal GDS score (*n* = 78) ^h^	20 (26%)
Clinical Frailty Score (*n* = 76)	
Median (Interquartile range)	5 (4, 6)
ADL score deficits	
Median (Interquartile range)	0 (0, 2)
IADL score deficits	
Median (Interquartile range)	2.5 (1, 5)
New diagnoses (*n* = 84) ^i^	
Cognition	55 (65%)
Cardiac	10 (12%)
Depression	7 (8%)
Neurological	6 (7%)
Renal	3 (4%)
Endocrine	2 (2%)
Respiratory	1 (1%)
Type of specialist physician referrals (*n* = 24) **^j^**	
Psychiatry	6 (25%)
Cardiology	4 (17%)
Ophthalmology	4 (17%)
Palliative care	3 (13%)
Neurology	3 (13%)
Urology	1 (4%)
Rheumatology	1 (4%)
Thromboembolism clinic	1 (4%)
Urogynecology	1 (4%)
Medication changes made	47 (47%)
Tests requested	
Labs	47 (47%)
Imaging	41 (41%)
Cardiac tests	18 (18%)
EMG	1 (1%)

Abbreviations: SD, standard deviation; MOCA, Montreal Cognitive Assessment; RUDAS, Rowland Universal Dementia Assessment Scale; MMSE, Mini-Mental State Examination; GDS, Geriatric Depression Scale; ADL, activities of daily living; IADL, instrumental activities of daily living; EMG, electromyography; ^d^ A separate analysis was conducted to exclude patients whose time delays were due to the patient/patient’s family rebooking, the patient being hospitalized during the time of appointment or clinic closure; ^e^ 26 patients had two reasons for referral; 12 patients had three reasons for referral; ^f^ Other reasons for referral included tremors, poor oral intake, chronic pain, sleep disturbance, difficulty in coping, dizziness and hearing loss; ^g^ Occupational therapy pre-assessments were replaced with nursing pre-assessments, and therefore only 54 patients were eligible to have an occupational therapy pre-assessment, and 46 patients were eligible for a nursing pre-assessment; ^h^ 3 patients completed the GDS with a normal/negative result but no score was documented on the consult, those respective patients were given a score of 3 (average score of GDS normal/negative patients); ^i^ 17/65 patients received two or more new diagnoses; ^j^ 3/21 patients referral received two types of referrals.

**Table 3:** Cancer treatment status and characteristics post-CGA.

	Total
	(*n* = 100)
Continuation of treatment after CGA (*n* = 46)	35 (76%)
Continuation of treatment and started new treatment after CGA (*n* = 46)	3 (7%)
Only started a new treatment after CGA (*n* = 54)	29 (54%)
Patients not on treatment before or after CGA	22 (22%)
**Patients referred to determine suitability for treatment**	
Recommendations for/against cancer treatment (*n* = 33) ^k^	
Suitable for treatment	16 (48%)
Suitable for treatment with modification	10 (30%)
Concern/against treatment	7 (21%)
Referring MD that followed through with the recommendation (*n* = 33)	31 (94%)
Cancer treatment status within 6 months after CGA (*n* = 33)	
On treatment	25 (76%)
Not on treatment	8 (24%)
Type of cancer treatment within 6 months after CGA (*n* = 33) ^l^	
Chemotherapy	17 (50%)
Immunotherapy	6 (18%)
Endocrine therapy	3 (9%)
Radiation	4 (12%)
Surgery	4 (12%)
Targeted therapy	1 (3%)
Dosing of treatment (*n* = 25)	
No change	9 (36%)
Reduced	10 (40%)
Increased	0 (0%)
N/A	6 (24%)
**Patients not referred to determine suitability for treatment**	
Cancer treatment status within 6 months after CGA (*n* = 67)	
On treatment	44 (66%)
Not on treatment	23 (34%)
Type of cancer treatment within 6 months after CGA (*n* = 50) ^m^	
Chemotherapy	11 (22%)
Immunotherapy	8 (16%)
Endocrine therapy	21 (42%)
Radiation	3 (6%)
Surgery	3 (6%)
Targeted therapy	4 (8%)
Dosing of treatment (*n* = 43)	
No change	24 (56%)
Reduced	13 (30%)
Increased	2 (5%)
N/A	4 (9%)

Abbreviation: CGA, comprehensive geriatric assessment; ^k^ Recommendations were only given to patients who were referred for treatment; ^l^ 7/25 patients CGA received two types of treatment, 1/25 patients received three types of treatment; ^m^ 6/44 patients received two types of treatment.

**Table 4:** ER visits and hospitalizations.

	Total
	(*n* = 100)
**ER visits within 6 months of CGA**	
Number of ER visits per patient	
Median (Interquartile range)	0 (0, 1)
Reason for first ER visit (*n* = 34) ^n^	
Cancer-related	12 (35%)
Comorbidity-related	12 (35%)
Treatment-related	4 (12%)
Infection-related	4 (12%)
Other	2 (6%)
Reason for second/third ER visit (*n* = 10) ^o^	
Cancer-related	3 (30%)
Comorbidity-related	3 (30%)
Treatment-related	1 (10%)
Infection-related	3 (30%)
**Hospitalizations within 6 months of CGA**	
Number of hospitalizations per patient	
Median (Interquartile range)	0 (0, 1)
Length of stay, days (*n* = 40)	
Median (Interquartile range)	7 (3, 12)
Reason for first hospitalization (*n* = 32) ^p^	
Cancer-related	16 (50%)
Infection-related	7 (22%)
Comorbidity-related	6 (19%)
Treatment-related	3 (9%)
Discharge location after first hospitalization (*n* = 30)	
Home with support services	10 (33%)
Home without support services	12 (40%)
Palliative care unit (Sunnybrook)	4 (13%)
Deceased	3 (10%)
Unknown	1 (3%)
Reason for second/third hospitalization (*n* = 10) ^q^	
Cancer-related	4 (40%)
Comorbidity-related	4 (40%)
Infection-related	1 (10%)
Treatment-related	1 (10%)
Discharge location after second/third hospitalization (*n* = 10) ^q^	
Home with support services	1 (10%)
Home without support services	3 (30%)
Rehab facility	2 (20%)
Deceased	4 (4%)

Abbreviations: CGA, comprehensive geriatric assessment; ER, emergency room; ^n^ 1/33 patients had two visit reasons; ^o^ 2/7 patients had two reasons for visit (second/third visit); ^p^ 1/30 patients had two reasons for hospitalization; ^q^ two patients had three hospitalizations.

## Data Availability

The data presented in this study may be available upon request from the corresponding author and authorization is required from the IRB in order to maintain and respect the confidentiality and privacy of this information.
